# Clinical and MRI Correlates of β‐Amyloid Load Inconsistent With Its Presumed Neurotoxicity in Cognitively Healthy Ageing

**DOI:** 10.1111/jnc.70241

**Published:** 2025-09-23

**Authors:** Pavel Filip, J. Riley McCarten, Laura Hemmy, Jillian Crocker, Michael Wolf, Jeromy Thotland, Zuzan Cayci, Todd Kes, Shalom Michaeli, Melissa Terpstra, Silvia Mangia

**Affiliations:** ^1^ Center for Magnetic Resonance Research (CMRR) University of Minnesota Minneapolis Minnesota USA; ^2^ Department of Neurology Charles University, First Faculty of Medicine and General University Hospital Prague Czech Republic; ^3^ Department of Cybernetics Czech Technical University in Prague Prague Czech Republic; ^4^ Geriatric Research, Education and Clinical Center Veterans Affairs Medical Center Minneapolis Minnesota USA; ^5^ Department of Neurology University of Minnesota Medical School Minneapolis Minnesota USA; ^6^ Department of Psychiatry University of Minnesota Medical School Minneapolis Minnesota USA; ^7^ Department of Radiology University of Minnesota Minneapolis Minnesota USA

**Keywords:** APP, cognition, general fitness, healthy ageing, quantitative MRI, β‐amyloid

## Abstract

Cognitively healthy ageing and its conceptual counterpart, dementia, have long garnered much interest in the research community, the broader public and regulatory bodies alike. Although β‐amyloid deposition is widely regarded as the principal neuropathological hallmark of Alzheimer's disease, its precise role in the causal chain of cognitive decline remains under debate. Applying strict criteria to define neurocognitive health, a selection of 35 participants aged over 60 years was drawn from the Human Connectome Project—Ageing. The evaluation of both cognitive and physical fitness, and comprehensive magnetic resonance imaging (MRI) protocol, encompassing diffusion‐weighted imaging, T1w/T2w ratio, resting‐state functional MRI and arterial spin labelling, were combined with an additional 18F‐florbetaben scan to evaluate β‐amyloid load. Strikingly, β‐amyloid load failed to adhere to the transcription patterns of amyloid precursor protein in all surveyed areas but the entorhinal cortex. Moreover, it was associated with either higher cognitive performance, general fitness, cerebral tissue integrity and cerebral perfusion, or had no discernible impact. This pilot study adds to the growing body of evidence that questions the significance attributed to β‐amyloid build‐up and the mechanisms of its accumulation in the ageing brain. The results invite a re‐evaluation of established theories on β‐amyloid build‐up neurotoxicity at low concentrations as observed in this cohort. Future investigations should focus on recruiting larger populations to ascertain whether a specific threshold of β‐amyloid build‐up precipitates cognitive decline or whether β‐amyloid accumulation, in fact, serves as a protective mechanism that ultimately fails.

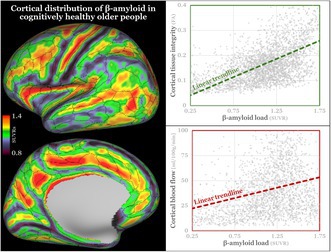

## Graphical abstract

At low levels, β‐amyloid—a protein commonly present in brains of patients with Alzheimer's disease – appears to actually support overall tissue integrity, blood flow, brain function and cognitive abilities, or at least not to cause harm. Our results suggest that β‐amyloid might initially build up as part of the brain's protective response and that the symptoms of Alzheimer's disease only develop when this protective mechanism eventually fails.

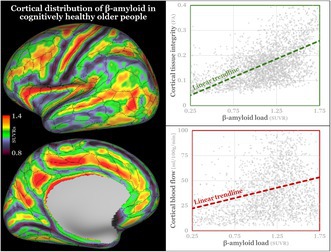

**Abbreviations**
AD: Alzheimer's disease
APP: amyloid precursor protein
BMI: body mass index
CBF: cerebral blood flow
CT: computer tomography
DTI: diffusion tensor imaging
DWI: diffusion weighted imaging
FA: fractional anisotropy
fALFF: fractional amplitude of low‐frequency fluctuations
FDR: false discovery rate
HCP‐A: Human Connectome Project‐Ageing
IVF: intracellular volume fraction
IWG: International Working Group
MD: mean diffusivity
MNI: Montreal Neurological Institute
MRI: magnetic resonance imaging
mRNA: messenger ribonucleic acid
NIA‐AA: National Institute on Ageing and Alzheimer's Association
NIH: National Institutes of Health
NODDI: neurite orientation dispersion and density imaging
ODI: orientation dispersion index
PET: positron emission tomography
ReHo: regional homogeneity
ROI: region of interest
rs‐fMRI: resting‐state functional magnetic resonance imaging
SUVR: standardized uptake value ratio
T1w: T1‐weighted
T2w: T2‐weighted
TE: echo time
TI: inversion time
TR: repetition time
wDeCe: weighed degree centrality
WM: white matter
WVF: free water volume fraction

## 1 Introduction

The concept of healthy ageing generally encompasses the absence of cognitive and motor frailty, pain and disabilities as a whole. The perception of its significance has been on a steady rise given the worldwide growth of the population over the age of 60 and substantial increase in average life expectancy in recent decades. Members of this age group have repeatedly voiced their concerns about the prospect of memory loss as one of the most feared aspects of ageing (Krivanek et al. 2021). Nonetheless, “typical” ageing processes, as envisioned by current theories, are associated with brain alterations ultimately leading to the decline of neurophysiological functions and increased susceptibility to a specific group of pathologies characterised by the aggregation of misfolded proteins (Martin et al. 2021). Decades of asymptomatic cumulation of such pathological changes may actually precede the conjectural breach of the cerebral reserve threshold and clinical manifestations of impairment (Driscoll and Troncoso 2011). The most common notional spectre for cognitive disruption is the deposition of β‐amyloid and aggregates of hyperphosphorylated tau protein, pathologies ultimately associated with Alzheimer's disease (AD) (Braak et al. 1993).

The amyloid cascade hypothesis postulates β‐amyloid accumulation as the first step of AD, forming in a sequential cleavage process of the amyloid precursor protein (APP) (Mucke and Selkoe 2012). The self‐assembled, misfolded β‐amyloid oligomer with hypothesised prion‐like properties (Walker et al. 2016) is then postulated to exert neurotoxic effects on several levels (Cohen et al. 2013; Mucke and Selkoe 2012). β‐sheets of β‐amyloid fibrils constitute then the principal targets of β‐amyloid imaging tracers employed alongside positron emission tomography (PET) (Maschio and Ni 2022). Their development, enabling non‐invasive detection and quantification of brain β‐amyloid deposition in vivo, represented a breakthrough in aetiological considerations in the differential diagnostic procedures in patients with cognitive complaints. Nonetheless, β‐amyloid PET scans have repeatedly indicated elevated levels in up to one‐third of individuals over 65 years of age devoid of major cognitive problems (Sperling et al. 2011).

The clinical implications of this finding remain a matter of dispute even at the level of two established diagnostic guidelines for AD. While both National Institute on Aging and Alzheimer's Association (NIA‐AA) (Petersen et al. 2021) and International Working Group (IWG) criteria (Dubois et al. 2021) acknowledge β‐amyloid and tau deposition as neuropathogenic hallmarks of AD, there is a fundamental disagreement on their relevance in cognitively unimpaired individuals. IWG criteria consider β‐amyloid and tau to be of ‘low predictive accuracy’ for the development of cognitive symptoms and classify such individuals as ‘at risk for progression to AD’, contrary to the more pessimistic designation of ‘preclinical AD’ as recommended by NIA‐AA. Long‐term follow‐up has associated β‐amyloid deposition in ‘healthy’ individuals with future cognitive decline (Donohue et al. 2017), albeit at levels below the minimal clinically important differences (Borland et al. 2022). Even in the largest relevant study to date (Ossenkoppele et al. 2022), the presence of β‐amyloid without tau was associated with similar rates of progression to mild cognitive impairment (MCI) and dementia as in participants devoid of any such deposition. Even when combined with positive tau findings, about a half of participants retained intact cognition after an average of 3.5 years of follow‐up.

Therefore, disentangling processes of genuine pathognomonic significance from those possibly inconsequential or even protective is an indispensable step in delineating the constituents of healthy brain ageing. To this end, we leveraged the uniquely comprehensive database of the Human Connectome Project‐Ageing (HCP‐A)—a research endeavour specifically designed to assess ageing, with an extensive magnetic resonance imaging (MRI) protocol and rigorous evaluation of both mental and physical fitness. We further restricted the standard inclusion criteria of the project to select only individuals over the age of 60 deemed cognitively intact based on history and objective evaluation by a neurologist and neuropsychologist specialised in AD, who then underwent an additional 18F‐florbetaben PET examination. The primary aim was to compare the spectrum of β‐amyloid load estimated based on β‐amyloid tracer binding, without hard dichotomisation into positive and negative classification based on empirical clinical thresholds, across preselected regions of interest (ROIs) relevant for general cognitive performance and/or AD pathology as envisioned in Braak's stages (Braak et al. 1993), in relation to the following:

– Aim 1: spatial distribution of APP messenger ribonucleic acid (mRNA) expression (Gryglewski et al. 2018) to cross‐validate the findings against the presumed source of β‐amyloid and to extrapolate eventual spread of β‐amyloid into areas with low APP mRNA expression, thereby indicating its limited local production;
– Aim 2: relevant demographic and clinical measures, encompassing physical and/or mental condition:
  ○ Age.
  ○ General fitness (grip strength, endurance in walking test and body mass index (BMI)).
  ○ Cognitive performance (crystallised and fluid cognition).
– Aim 3: MRI measures as proxies of underlying central nervous system histology and/or function to characterize tissue with higher β‐amyloid load further and to evaluate these MRI metrics as indirect, surrogate markers of β‐amyloid:
  ○ T1‐weighted/T2‐weighted signal (T1w/T2w) ratio for myelin content (Glasser and Van Essen 2011).
  ○ Fractional anisotropy (FA) and mean diffusivity (MD) for oedema and cellular integrity (Beaulieu 2014).
  ○ Orientation dispersion index (ODI), free water volume fraction (WVF) and intracellular volume fraction (IVF) as combined proxies of myelination, cellular integrity and inflammation (Kamiya et al. 2020).
  ○ Cerebral blood flow (CBF).
  ○ Resting‐state functional MRI (rs‐fMRI) metrics as weighted degree centrality (wDeCe), regional homogeneity (ReHo) and fractional amplitude of low‐frequency fluctuations (fALFF) as proxies of general neuronal activity and connectivity.

We hypothesised that in cognitively intact individuals, β‐amyloid distribution would exhibit a high level of spatial correspondence with APP transcriptome, reflecting mere localised β‐amyloid production without substantial subsequent spread via other routes. Furthermore, we postulated that participants exhibiting superior physical fitness and cognitive performance would possess lower β‐amyloid loads and that higher β‐amyloid presence would be associated with reduced tissue integrity (higher MD, lower FA, higher ODI and lower T1w/T2w ratio), lower CBF and diminished functional activity (lower wDeCe, ReHo, fALFF).

## 2 Methods

### 2.1 Study Participants

The HCP‐A cohort acquired at the University of Minnesota, USA, from 2017 to 2022, was screened for eligible participants. For the general project information, including the full list of inclusion and exclusion criteria, please see (Bookheimer et al. 2019). Briefly, the following HCP‐A exclusion criteria were of relevance and/or not superseded by the additional criteria employed in the presented study:

Across the participants' lifetime:

– Any neurological disease (with the exception of back pain and migraines not requiring prescription medication).
– Major psychiatric disorder.
– Brain surgery.
– Severe head injury.
– Hospitalization for two or more days due to alcoholism or drug dependence.
– Rheumatoid arthritis.
– HIV infection.
– Lupus or any other condition necessitating prolonged use of steroids or other immunosuppressants.
– Macular degeneration in individuals under 80 years of age.
– Known genetic disorder.

Within the year preceding enrolment:

– Diagnosis of thyroid dysfunction and/or alteration in thyroid medication dosage.
– Myocardial infarction.

At the time of the study:

– Diabetes diagnosed within the preceding three months, or poorly controlled diabetes (glycated haemoglobin 1c > 7.0).
– Pregnancy.
– Claustrophobia.
– Migraine within 72 h of the study visit.
– Uncontrolled blood pressure (> 170/100 mmHg) or ongoing optimisation of antihypertensive medication.
– Severe pulmonary, hepatic, renal, or cardiac disease, or any other major organ failure.

The additional, more stringent exclusion criteria for the presented study were as follows:

– Any procedure requiring general anaesthesia and/or associated with perioperative complications potentially affecting cognitive function within six months prior to study inclusion.
– New neurological problems that developed between the HCP‐A MRI acquisition and enrolment, including previously undiagnosed pathological findings at HCP‐A MRI scans.
– Cognitive performance considered outside normal range for demographic characteristics based on neuropsychological data available in the HCP‐A protocol (test performances over 1.5 standard deviations below normative data).
– Abnormal neurologic status based on a detailed medical history and comprehensive neurological examination (Christensen et al. 1992; McCarten 2013).

In total, 178 HCP‐A participants at the age of 60 or more were pre‐screened, 37 participants were referred for specialised neurological examination, and ultimately, 35 participants were enrolled in this study (see Figure S1 for flowchart with more information). A separate analysis of data from a partly overlapping cohort was previously included in a study aimed at characterising microstructural patterns of grey matter distinguishing cognitively healthy from typical ageing (Filip et al. 2025).

Institutional review board (IRB) approval from the IRB of the University of Minnesota was obtained (approval reference numbers STUDY00001744 and SITE00000071), and all the participants provided their written informed consent. The study was not pre‐registered.

### 2.2 MRI Data Acquisition and Pre‐Processing

The full HCP‐A protocol, including the clinical tests, was extensively described and discussed (Harms et al. 2018). This study utilised the following MRI acquisitions:

– T1w: multi‐echo MPRAGE sequence, 0.8 mm isotropic resolution, repetition time (TR) 2500 ms, inversion time (TI) 1000 ms, echo time (TE) 1.8/3.6/5.4/7.2 ms.
– T2w scans: SPACE sequence, 0.8 mm isotropic resolution, TR 3200 ms, TE 564 ms.
– Diffusion weighted imaging (DWI): 1.5 mm isotropic resolution, TR 3222 ms, TE 89.20 ms, multi‐band (MB) acceleration factor 4, *b*‐values of 1500 and 3000 s/mm^2^, 93 directions with 7 additional b0 images, two acquisitions with opposite phase‐encoding polarity in the antero‐posterior axis.
– rs‐fMRI acquisition: 2 mm isotropic resolution, TR 800 ms, TE 37 ms, MB8, 488 volumes, two acquisitions with opposite phase encoding polarity in the antero‐posterior axis, including two spin‐echo echo‐planar imaging scans with opposite phase encoding directions for field inhomogeneity‐related distortion correction. Subject underwent the acquisition with open eyes, fixating on a single point.
– CBF—pseudo‐continuous arterial spin labelling, labelling duration 1500 ms, five post‐labelling delays of 200 ms (6 control/label pairs), 700 ms (6 pairs), 1200 ms (6 pairs), 1700 ms (10 pairs), 2200 ms (15 pairs); 2.5 mm isotropic voxel resolution, TR 3580 ms, TE 19 ms.

The MRI processing pipeline was based largely on the HCP minimal preprocessing pipeline (Glasser et al. 2013), FreeSurfer version 6.0, including the optional gradient non‐linearity corrections and susceptibility‐induced B0 field deviation correction applied to the structural scans. Subsequently, the diffusion tensor parameters (FA and MD) were extracted using constrained weighted linear least‐squares fit (Veraart et al. 2013). Non‐Uniformity and Distortion Correction for Diffusion Imaging (NODDI) parameters (IVF, WVF, ODI) were calculated using the NODDI toolbox (http://nitrc.org/projects/noddi_toolbox). rs‐fMRI analysis after the HCP minimal preprocessing pipeline followed the HCP rs‐fMRI pipeline (Smith et al. 2013), and the AFNI package was utilised to calculate wDeCe with a sparsity threshold of 0.1 (Craddock and Clark 2016), ReHo as Kendall's W over 28 neighbouring voxels (Zang et al. 2004), and fALFFF within the frequency range between 0.01 and 0.1 Hz (Zang et al. 2007). CBF values were extracted using a previously validated two‐compartment model at each post‐labelling delay using least‐squares fitting (Juttukonda et al. 2021). All outputs described above were reviewed visually by a trained operator (P.F.). Framewise root‐mean‐squared voxel displacement was assessed in multi‐volume data with an exclusion framewise motion threshold at the level of two voxels.

For full PET acquisition procedure, see Data S1. Briefly, the participants were administered a single dose of 8.1 mCi±10% 18F‐florbetaben followed by a combined brain PET/computer tomography (CT) scan after 60 min. In the following step, rigid body coregistration of the CT scan to the T1w scan aligned to the anterior commissure—posterior commissure line as produced by the HCP pipeline (native HCP space) was used for the initialisation of further boundary‐based registration of PET data to the native HCP space. Voxel‐wise standardised uptake value ratios (SUVRs) were calculated based on average PET data intensities over the FreeSurfer‐derived cerebellar mask. For the purposes of comparison with the APP transcriptome atlas (Gryglewski et al. 2018) (see Figure S2 for visualisation), the β‐amyloid SUVR maps (β‐amyloid load) were spatially normalised utilising the warp to the common Montreal Neurological Institute (MNI) space previously generated by the HCP pipeline.

The following bilateral ROI masks were derived from the automatic FreeSurfer labelling, both in native HCP space and in MNI space, in each subject (see Figure S3):

– Entorhinal cortex as the closest FreeSurfer approximation for the transentorhinal Braak stage I–II;
– Combined mask for subcortical limbic system structures (hippocampi, amygdalae and accumbens nuclei); whole temporal lobe cortex as the approximation for the limbic Braak stage III–IV;
– Rostral anterior cingulate cortex; combined mask for praecunei and posterior cingulate cortices as areas commonly considered in clinical β‐amyloid PET scan evaluations;
– Whole cortex; whole white matter (WM); and major basal ganglia structures (caudate, putamen, pallidum) for overview of basic characteristics over general, large ROIs. WM ROI was considered only for β‐amyloid load versus MRI metric correlation due to dubious clinical significance of off‐target 18F‐florbetaben binding in WM and lack of APP mRNA expression data in the APP transcriptome atlas.

Inverse registration matrices from the native HCP space to individual MRI metric and PET image spaces were used to coregister the ROI masks to the aforementioned lower resolution spaces, with mask thresholding at the probability of inclusion in the relevant ROI in the lower resolution space at the level of at least 0.9 to mitigate partial volume effects. This approach yielded mask sizes ranging from about 50 voxels for smaller ROIs such as the entorhinal cortex to about 4000 voxels for large ROIs such as the whole cortex. Average β‐amyloid load and MRI metric values were then extracted over these coregistered masks in the native space of each image. Lastly, all the imaging parameters of interest and ROI masks were resampled to 2‐mm isotropic resolution in the native HCP space. Resampled 2‐mm isotropic resolution masks were again thresholded with 0.9 inclusion probability. Voxel‐wise Pearson's correlation coefficients between β‐amyloid load and individual MRI metrics were calculated over all the grey matter ROIs in this 2‐mm isovoxel native HCP space. For the APP transcriptome atlas, voxel‐wise Pearson's correlation coefficients with β‐amyloid load were calculated in the MNI space.

### 2.3 Clinical Metrics

We leveraged the National Institute of Health (NIH) Toolbox tests included in the HCP‐A protocol, specifically the age‐corrected standard scores to mitigate the effect of age in cross‐sectional analyses. For general fitness, dominant hand grip strength and 2‐min walk endurance test were considered, but since the latter was available only for 19 out of 35 participants due to limitations imposed in data acquisition protocols during the COVID‐19 pandemic, it was excluded from the main final Aim 2 analysis and provided only in a supplementary evaluation. For the evaluation of cognition, composite scores from the NIH Toolbox cognition battery were employed—fluid and crystallised cognition composite scores. Fluid cognition composite score is based on Flanker, Dimensional Change Card Sort, Picture Sequence Memory, List Sorting and Pattern Comparison tests. Crystallised cognition composite includes Picture Vocabulary Test and Oral Reading Recognition Test.

### 2.4 Statistical Approach

No formal sample size calculation was performed for this exploratory study. Basic demographic and clinical metrics were summarised as group averages with standard deviations. A supplementary comparison was also performed to assess eventual differences between groups deemed β‐amyloid positive and negative in the clinical reading, where a two‐sample two‐tailed Student's *t* test (for continuous variables) or *χ*2 (for categorical variables) was utilised, with False Discovery Rate (FDR) adjustment across all the presented comparisons (Storey 2002). No tests for outliers or imputations of missing values were performed.

One‐way one‐sample permutation‐based *t*‐test was used to evaluate whether absolute values of subject‐specific, Fisher‐transformed, voxel‐wise Pearson's correlation coefficients of β‐amyloid load with APP transcriptome atlas and individual MRI metrics was larger than 0.3 selected as the empirical medium effect size (Cohen 1988) (Aims 1 and 3). For Aim 2, Pearson's correlation coefficients of β‐amyloid load with demographic and clinical metrics was calculated, with permutation‐based statistical significance testing. Lastly, to assess similarity between the association of β‐amyloid load and MRI metrics with demographic and clinical parameters, correlation matrices of all the MRI metrics employed with demographic and clinical parameters were calculated, vectorised and correlated to the vectorised correlation matrix of β‐amyloid load and demographic and clinical parameters from Aim 3. Permutation‐based non‐parametric analysis as implemented in the Permutation Analysis of Linear Models package (Winkler et al. 2014), with 10,000 permutations and FDR correction (Storey 2002) over modalities within each aim was employed. Results were considered statistically significant at the predetermined alpha of 0.05.

## 3 Results

Thirty‐five HCP‐A participants (22 females, 13 males) met the stringent, expanded criteria and completed the 18F‐florbetaben PET scan. Their demographic and clinical parameters are presented in the Table 1. The enrolled cohort generally exhibited levels of physical fitness and cognitive performance above the age‐specific averages (values > 100 for age‐corrected standard scores), with expectable associations between the individual demographic and clinical variables (see Table S1). Based on the clinical reading of 18F‐florbetaben PET scans, 24 participants were considered β‐amyloid negative, while 11 were deemed positive. The comparison of clinical metrics of interest between these two groups failed to show any statistically significant differences (see Table S2).

**TABLE 1:** Basic demographic and clinical characteristics of the cohort.

	Whole cohort	Number of participants with available data
Age (years)	73.17 [9.07]	35
Sex (females/males)	22/13	35
Body mass index (BMI) (kg.m^−2^)	26.56 [4.45]	35
Education (years)	16.31 [2.23]	35
BAPL score (number of participants with score 1/2/3)	24/6/5	35
NIH toolbox cognitive function battery (CFB)		
Fluid cognitive abilities (age‐corrected standard score)	112.71 [15.28]	35
Crystallised cognitive abilities (age‐corrected standard score)	109.85 [11.54]	34
NIH toolbox motor domain		
Grip strength, dominant hand (age‐corrected standard score)	101.81 [15.49]	31
2‐min walk endurance test (age‐corrected standard score)	116.00 [10.92]	19

*Note:* Data presented as average [standard deviation] and number of participants with available datapoints, unless specified otherwise.

**FIGURE 1:**
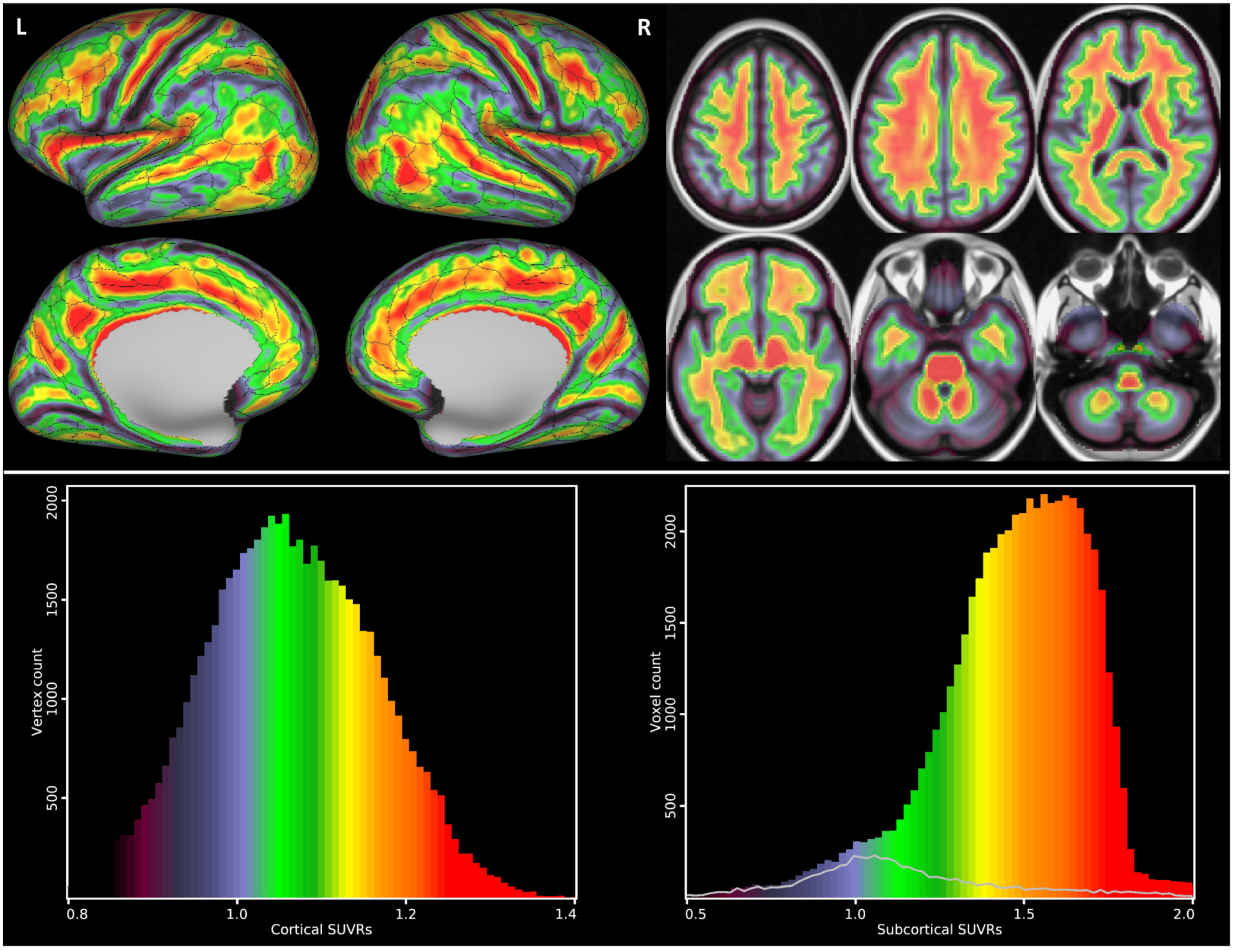
Average β‐amyloid standardised uptake value ratio (SUVR) map over the full cohort of 35 participants: Cortical surface reconstruction at the left side with overlaid Human Connectome Project (HCP) cortical parcellation borders (Glasser et al. 2016) and subcortical structures in Montreal Neurological Institute (MNI) space at the right side shown in 6 slices *z* = 48, 29, 10, −28, −47 (MNI coordinate system). Colour‐coding provided in histograms below. Laterality convention where the right side of the figure corresponds to the right side of the brain is used. The lower part of the figure shows histograms of cortical and subcortical SUVR maps (*y* axes as vertex and voxel count, respectively) to visualise skewness (symmetric for grey matter structures and negative for white matter). Colour‐coding corresponds to the scale utilised in the upper part of the figure. Grey‐line histogram subsection in the subcortical SUVR histogram corresponds to subcortical grey matter structures (excluding the cerebellum). L, left; R, right.

Figure 1 and Table S3 present the cortical and subcortical whole‐group average β‐amyloid, showing higher β‐amyloid accumulation in expectable regions such as the cingulate, precuneus and lateral temporal cortex, but also select frontal cortical regions such as the frontal opercular area, areas 4, 6 and 8, inferior frontal junction area and insula, exhibiting generally good lateral similarity and remarkably high correspondence to the overlaid HCP cortical parcellation, even to the point of following regions newly defined by HCP (Glasser et al. 2016). The subcortical regions were generally dominated by the non‐specific, off‐target 18F‐florbetaben binding in WM, as evidenced also in the histogram in Figure 1.

### 3.1 Aim 1—β‐Amyloid Load Versus APP Transcriptome

**FIGURE 2:**
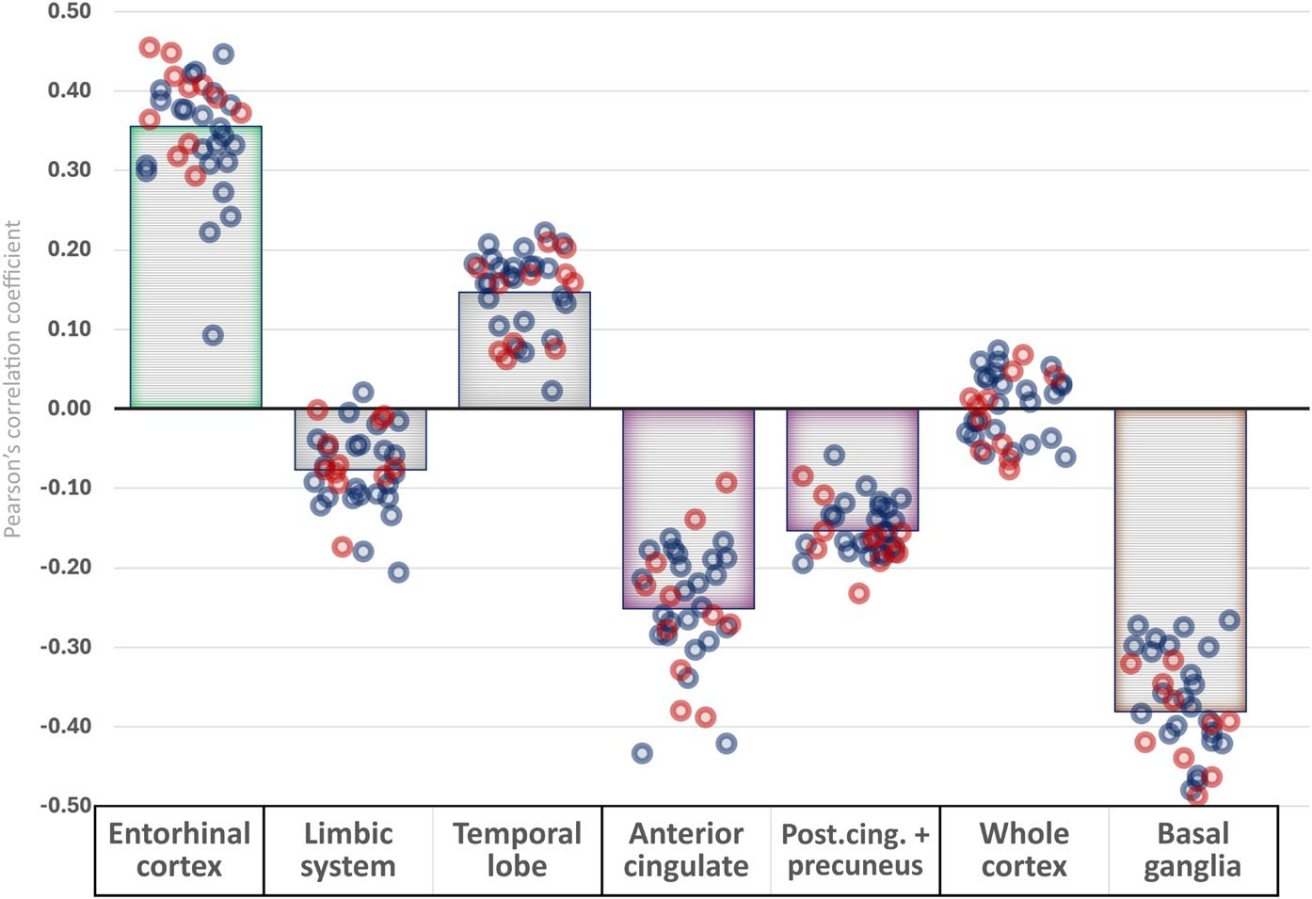
Correlation of amyloid precursor protein (APP) mRNA expression intensity and β‐amyloid load (standardised uptake value ratios [SUVRs]) for the main regions of interest. The bars represent full cohort averages. Individual subject values are shown as blue (negative clinical reading of florbetaben scan) and red (positive clinical reading) circles, with random jitter along the *x* axis for better legibility. All areas exhibit substantial clustering of individual values, without major differences between individuals with positive and negative clinical reading. See Table S3 for more information. post. cing., posterior cingulate.

Only entorhinal cortex exhibited positive voxel‐wise correlation statistically significantly higher than 0.3 (see Figure 2, Table S3). On the other hand, the other ROIs showed either no correlation or anti‐correlation in the range of low effect size, with basal ganglia being the only region reaching preselected statistical significance.

### 3.2 Aim 2—β‐Amyloid Load Versus Demographic and Clinical Parameters

**FIGURE 3:**
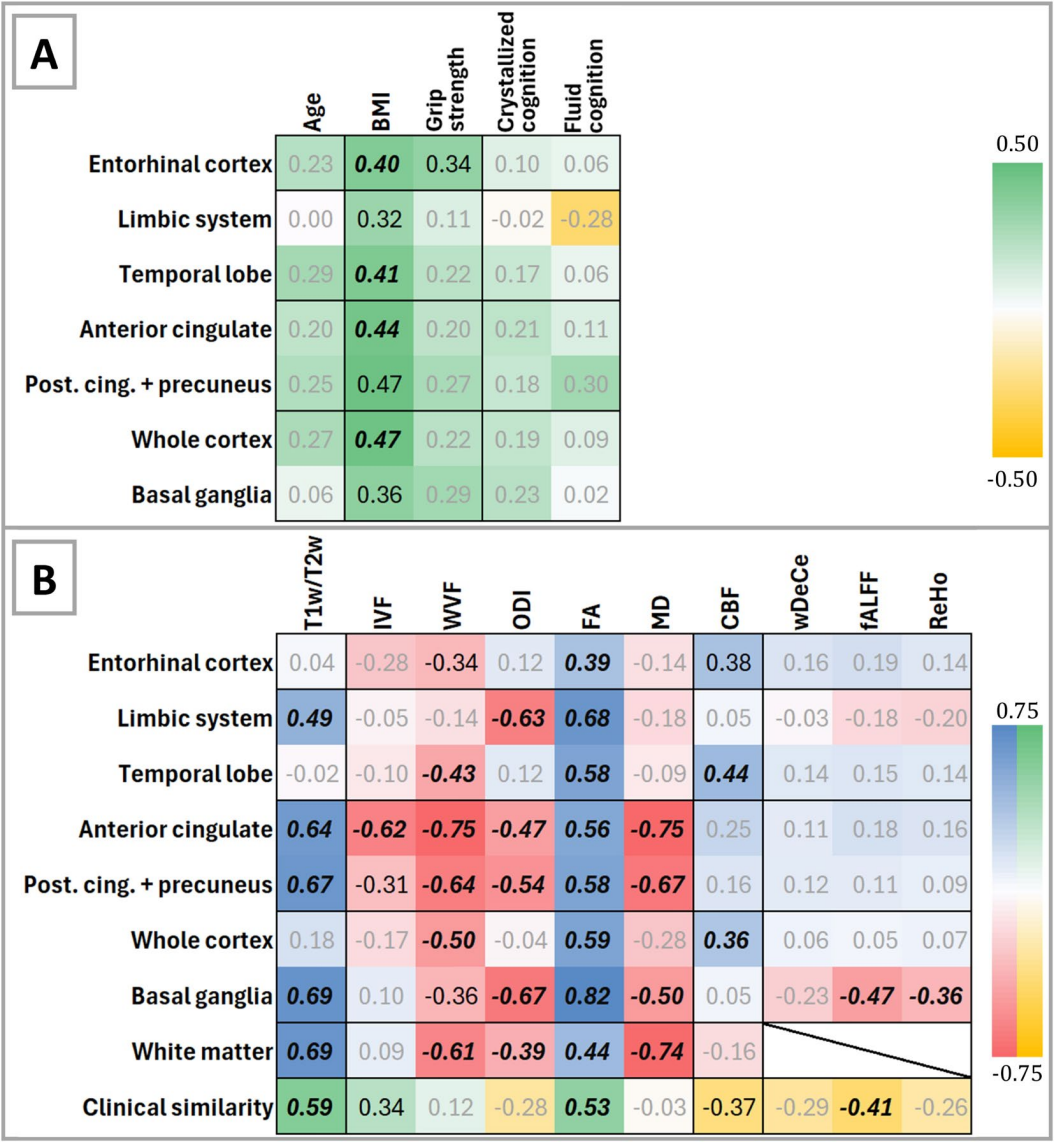
Correlation of β‐amyloid standardised uptake value ratios (SUVRs) with (A) demographic and clinical metrics; (B) magnetic resonance imaging (MRI) metrics over preselected regions of interest. Yellow‐green and red‐blue scale visually show Pearson's correlation coefficients; absolute correlation coefficient values below 0.3 are provided in grey‐coloured numbers, above 0.3 in black colour and statistically significantly above 0.3 (false discovery rate corrected *p*‐values < 0.05) in bold italics to enhance visibility. See also Tables S4, S6 and S7. BMI, body mass index; CBF, cerebral blood flow; FA, fractional anisotropy; fALFF, fractional amplitude of low‐frequency fluctuations; IVF, intracellular volume fraction; MD, mean diffusivity; ODI, orientation dispersion index;m; Post.cing., posterior cingulate; ReHo, regional homogeneity; T1w/T2w, T1‐weighted/T2‐weighted ratio; wDeCe, weighted degree centrality; WVF, free water volume fraction.

As evident in Figure 3A, β‐amyloid load over ROIs exhibited no correlation or generally positive correlations, albeit in the area of low effect size with all the metrics of interest, including age, with the exception of fluid cognition for limbic system β‐amyloid load. The only results in the medium effect range were provided for BMI (with statistically significant results over major areas like whole cortex, but also temporal lobe, entorhinal and anterior cingulate cortex) and for grip strength (see also Table S4 for FDR corrected *p* values). Since these results not only failed to confirm our initial hypothesis, a supplementary analysis of non‐dominant hand grip strength, 2‐min walk endurance test and 4‐m gait speed test was added (see Table S5), showing again either no correlation or mildly positive correlations (low or medium effect size).

### 3.3 Aim 3—β‐Amyloid Load Versus MRI Metrics

Full DWI acquisition was not available for 2 participants, and 1 participant failed to complete CBF measurement. Ergo, the analyses of relevant metrics were based on 33 and 34 individual datasets, respectively. The analyses of non‐affected MRI metrics consider the whole cohort. Several microstructural metrics exhibited voxel‐wise correlations over multiple ROIs with β‐amyloid load (see Figure 3B, Tables S6 and S7), with statistically significant levels over most or even all ROIs for T1w/T2w ratio and FA, respectively, including the clinical similarity assessment (see also Table S8). On the other hand, MD, WVF and ODI exhibited generally negative correlations. The correlation between CBF and β‐amyloid load was generally positive, reaching medium effect size in entorhinal cortex (although not statistically significant); and temporal lobe and whole cortex (statistically significantly higher than 0.3). However, fMRI metrics failed to show any significant association with β‐amyloid load, with the exception of basal ganglia.

## 4 Discussion

β‐amyloid, often regarded as a portent of the future collapse of cognitive abilities and a diagnostic hallmark of AD pathology, has been the focus of the present study, which extended upon the unique, multimodal dataset of the HCP‐A, involving more than 6 h of examination per participant across multiple sessions. Nonetheless, the findings generally fail to confirm both the presumed APP‐related early mechanisms of β‐amyloid accumulation and its putative neurotoxic properties at lower concentrations, given the strong positive association of β‐amyloid load with markers of microstructural tissue integrity.

Although considerable research efforts have substantially advanced our understanding of β‐amyloid pathophysiology and imaging, a definitive account of how β‐amyloid assemblies—whether fibrils or protofibrils—contribute to pathological processes remains elusive (Maschio and Ni 2022). A once‐prevalent hypothesis proposed that the formation of β‐amyloid plaques compromises synaptic function, thereby precipitating the hallmark memory impairments characteristic of symptomatic AD (Selkoe and Hardy 2016). However, the extent of β‐amyloid infiltration within various brain regions has repeatedly failed to provide consistent correlations with the progression and severity of clinical symptoms (Rischel et al. 2023), in stark contrast to hyperphosphorylated tau protein (Ossenkoppele et al. 2022). Lessons from animal studies also provided conflicting results. Although older non‐human primates develop β‐amyloid deposits sharing the human amino acid sequence, they fail to manifest the characteristic clinical signs seen in humans (Rosen et al. 2016). Perhaps most importantly, numerous anti‐β‐amyloid drugs in over 30 different clinical trials on mild to moderate AD, MCI and even cognitively intact individuals with positive β‐amyloid PET scans yielded substantial reduction in cerebral β‐amyloid burden without conferring major cognitive benefits (Abanto et al. 2024).

Our initial hypotheses were grounded on seemingly sound presumptions regarding established risk factors of future cognitive decline: higher BMI, even though contested and likely oversimplified consideration (Anstey et al. 2011; Xu et al. 2011), poor physical fitness (Kulmala et al. 2014; Kurl et al. 2018) and low cognitive reserve (Nelson et al. 2021; Wang et al. 2017). Nonetheless, despite the expectable directions of the associations between individual age, BMI, general fitness and cognitive performance parameters (see Table S1), the direction of β‐amyloid load correlations with general fitness and cognitive performance was largely positive, in stark contrast to our initial expectation. The mildly positive, albeit statistically not significant age correlation with β‐amyloid load is generally in line with previous reports (Jansen et al. 2015). Strictly statistically speaking, only BMI displayed significant results; the remaining metrics yielded small effect sizes when assessed based on empirical recommendations by Cohen for situations with unknown field‐specific estimates (Cohen 1988). However, recent guidelines grounded in a meta‐analysis of gerontological studies suggested more lenient Pearson's thresholds of 0.10, 0.20 and 0.30 for low, medium and large effect sizes, respectively (Brydges 2019). Under these criteria, our findings would shift to medium and large levels, despite our cohort size being insufficient for drawing conclusive inferences regarding these correlations.

Furthermore, the absence of a positive association between APP mRNA expression and actual β‐amyloid load in areas other than the entorhinal cortex, culminating with a statistically significant negative correlation in the basal ganglia, warrants attention. The cross‐sectional data exhibited generally reasonable clustering around stable levels for all examined ROIs. While the prevalence of β‐amyloid positivity mirrors normal population expectations (Jansen et al. 2022; Sperling et al. 2020) despite our stringent inclusion criteria, BAPL positivity in this cognitively healthy cohort conferred no discernible inter‐group differences not only in clinical or neuropsychological findings (see Table S2) but also in the correlation between APP mRNA expression and β‐amyloid load (see Figure 2, Table S3). All in all, these similarities preserve the central interpretation of β‐amyloid tracer uptake in cognitively intact individuals as a continuum instead of hard binarisation based on clinical readings developed for symptomatic cases.

Overall, these results might be interpreted as indirect signs of the non‐local origin of β‐amyloid in areas other than the entorhinal cortex. While hypotheses proposing prion‐like mechanisms of β‐amyloid spread have been gaining traction, later stages of pathology translating into apparent clinical symptomatology were surmised in this cascade (Walker et al. 2016). The suggestion of this process in cognitively intact individuals would be speculative at best. An alternative explanation may involve the previously proposed interplay of compromised cerebral microcirculation and subsequent β‐amyloid buildup (Zlokovic 2011). A dysfunctional brain–blood barrier could facilitate the infiltration of neurotoxic molecules, exacerbate regional hypoxia due to impaired capillary flow, and also impede the clearance and transvascular transport of metabolic waste (Sweeney et al. 2018), culminating in β‐amyloid accumulation.

Indeed, CBF exhibited a positive association with β‐amyloid load over whole and temporal lobe cortical ROIs, but not in the basal ganglia—the area that displayed the most pronounced divergence between APP transcriptome and β‐amyloid accumulation. Such positive correlations are in line with previous reports for early stages of the AD spectrum (Daneshpour et al. 2025), although several studies have documented a subsequent reversal and reduction in global CBF in later phases (Zhang et al. 2021), underscoring continuing discrepancies in reported outcomes. Meanwhile, rs‐fMRI metrics yielded no observable functional alterations linked to βß‐amyloid load contrary to the initial hypothesis postulating decreased connectivity and local activity due to β‐amyloid‐related disruption of neuronal function. All in all, both CBF and rs‐fMRI metrics seem to add further credence to the emerging picture of the absence of neurotoxic effects of β‐amyloid in the presented cohort.

The microstructural measures employed in this study generally aligned far more closely with the data obtained from 18F‐florbetaben scans, with the highest correspondence achieved by FA and T1w/T2w ratio, even at the level of associations with demographic and clinical metrics of interest (see Figure 3B, Tables S4 and S5). In cortical regions, FA has been previously associated with unmyelinated neurites, mostly large calibre apical dendrites (Reveley et al. 2022), whereas T1w/T2w ratio has been associated with myelin content based on cytoarchitectonic cortical myelin maps (Glasser and Van Essen 2011) and multiple sclerosis research (Filip et al. 2020). Nonetheless, overzealous interpretation of any increase in these indices alongside β‐amyloid load as evidence of increased myelination or dendritic complexity would be premature. Microstructural measures offer only an indirect perspective of combined contrast drivers such as paramagnetic iron (Möller et al. 2019), cell membranes (Palombo et al. 2020) and myelin (Stüber et al. 2014), but remain highly pertinent for ageing research (Filip et al. 2023; Filip et al. 2025). Moreover, the inverse association of ODI with β‐amyloid load reinforces the notion of increased underlying structural intricacy. When seen in the light of other metrics also pointing to increasing tissue complexity, such as the reduction in MD and WVF with rising β‐amyloid load, the emerging picture again challenges the presumed neurotoxic properties attributed to β‐amyloid (Cohen et al. 2013; Mucke and Selkoe 2012). On the contrary, MRI data collectively indicate preserved functional features, enhanced blood perfusion and an overall tissue complexity increase, supporting the hypothesis that in cognitively intact individuals, relatively low β‐amyloid loads might assume a protective role (Rischel et al. 2023).

Nevertheless, the interpretation of these findings necessitates consideration of several key limitations. First and foremost, correlations per se do not imply causation. It is imperative to consider possible intermediates and external confounding factors in careful examination and further validation of β‐amyloid effects in clinically asymptomatic individuals. However, the methodological approach undertaken here enabled us to circumvent the binarization and over‐reliance on strict, arbitrary statistical thresholds. Given the extraordinary extent of data acquisition inherent to the complex evaluation of cognitively healthy ageing as presented in this study, integrating effect size metrics afforded a constructive framework for interpretation of our pilot data and recommendations for further research. Nonetheless, the modest cohort size precludes the reliable utilisation of feature selection mapping or other more advanced methods theoretically enabling more definitive statements on the interplay of effects of demographic, socioeconomic and environmental factors. Secondly, our outcomes are largely based on cross‐sectional comparisons in a cohort of limited size, precluding definitive conclusions. Even at the presented level, the study required multiple sessions at a research‐level MRI scanner, PET acquisition, motor assessment and examinations by several specialists in neuropsychology and cognitive neurology, followed by complex data processing. An ideal scenario would entail systemic follow‐up from midlife over several decades to assess the development of cognitive performance. However, such an undertaking would be prohibitively costly and challenging to implement. Nonetheless, should the current hypothesis of the absence of β‐amyloid neurotoxic effects at lower loads or even its protective properties (Rischel et al. 2023) gain further support, extensive investigations would be necessary to clarify the inconsistent associations, with immense clinical ramifications.

## 5 Conclusion

Not only did the presented results fail to demonstrate the originally hypothesised neurotoxic effects of β‐amyloid accumulation at low levels as seen in cognitively intact individuals; but on the contrary, β‐amyloid load either was positively correlated with cognitive performance, general fitness, cerebral tissue integrity, cerebral perfusion and rs‐fMRI metrics, or had no discernible impact. This study adds to the growing body of evidence challenging the significance attributed to β‐amyloid aggregation in the ageing brain, along with the mechanisms responsible for its deposition and invites a re‐evaluation of established theoretical paradigms. Future research should focus on recruiting far larger cohorts, preferably hundreds of subjects, including tau deposit measurements to determine whether there are indeed specific general or individual thresholds of β‐amyloid build‐up that would precipitate cognitive decline or whether β‐amyloid accumulation genuinely functions as a protective mechanism that ultimately fails, allowing for the breakthrough of clinical symptoms seen in Alzheimer's disease.

## Author Contributions

**Pavel Filip:** conceptualization, methodology, visualization, formal analysis, writing – original draft, software. **J. Riley McCarten:** writing – review and editing, investigation, methodology. **Laura Hemmy:** writing – review and editing. **Jillian Crocker:** writing – review and editing, data curation, project administration. **Michael Wolf:** writing – review and editing, data curation, project administration. **Jeromy Thotland:** writing – review and editing, investigation, software. **Zuzan Cayci:** writing – review and editing, investigation, methodology. **Todd Kes:** writing – review and editing, investigation, methodology. **Shalom Michaeli:** writing – review and editing, supervision, conceptualization. **Melissa Terpstra:** writing – review and editing, funding acquisition, resources. **Silvia Mangia:** writing – review and editing, funding acquisition, conceptualization, supervision, validation, resources.

## Ethics Statement

The study protocol was approved by the Institutional Review Board of the University of Minnesota (reference numbers STUDY00001744 and SITE00000071).

## Consent

Each subject provided a written informed consent in accordance with the Declaration of Helsinki.

## Conflicts of Interest

The authors declare no conflicts of interest.

## Peer Review

The peer review history for this article is available at https://www.webofscience.com/api/gateway/wos/peer‐review/10.1111/jnc.70241.

## Supporting information


**Data S1:** Flowchart documenting the pre‐screening process from the full HCP‐A database acquired at the University of Minnesota, USA from 2017 to 2022, stating relevant numbers of individuals and reasons for exclusion. “Declined participation” includes HCP‐A participants who explicitly declined the option to be contacted for follow‐up projects and HCP‐A participants who declined after being contacted with the offer for participation in this specific study. MoCA, Montreal Cognitive Assessment; MRS, magnetic resonance spectroscopy; TICS, Telephone Interview for Cognitive Status (the full study protocol included also MRS data which is not the subject of this analysis).
**Figure S2:** Amyloid precursor protein (APP) transcriptome atlas with cortical reconstruction at the left side with overlaid Human Connectome Project (HCP) cortical parcellation borders (Glasser et al. 2016) and subcortical grey matter at the right side over a T1‐weighted template. Colour scales represent log2 messenger ribonucleic acid expression intensity. Subcortical structures shown in 6 slices *z* = 23, 11, −1, −13, −25, −37 (Montreal Neurological Institute coordinate system) with focus on subcortical grey matter structures, since no white matter expression intensity data was available. High APP expression intensities depicted in lateral temporal cortices, anterior and posterior cingulate, precuneus, but also lateral parts of both putamina. On the other hand, low APP expression intensities are present in the right temporal cortex, lateral frontal cortices and the cerebellum. Laterality convention where the right side of the figure corresponds to the right side of the brain is used. See Table S4 for further anatomical and statistical information on significant regions. L, left; R, right.
**Figure S3:** Regions of interest in a “representative” subject. (A) Native resolution of the T1‐weighted space; (B) 2‐mm isotropic voxel resolution thresholded with 0.9 inclusion probability to mitigate partial volume effects in lower resolution scans as resting‐state functional MRI, cerebral blood flow and diffusion‐weighted imaging metrics. Colour coding as follows: entorhinal cortex—red; subcortical limbic structures—orange; temporal lobe cortex—yellow; anterior cingulate—cyan; posterior cingulate and precuneus—blue; basal ganglia—green. Whole cortex and whole white matter masks not depicted for clarity purposes. *Z* coordinates of slices 40, 34, 27, 21, 14, 18, 2, −5, −11, −18, −24, −30.
**Figure S4:** Average β‐amyloid standardised uptake value ratio (SUVR) map separately over the full cohort (35 participants), individuals with negative (24 participants) and positive clinical reading (11 individuals): cortical surface reconstruction at the left side with overlaid Human Connectome Project (HCP) cortical parcellation borders (Glasser et al. 2016) and subcortical structures in Montreal Neurological Institute (MNI) space at the right side shown in 6 slices *z* = 48, 29, 10, −28, −47 (MNI coordinate system). Colour‐coding provided in scales below. Laterality convention where the right side of the figure corresponds to the right side of the brain is used.
**Table S1:** Cross‐correlation matrix of individual demographic and behavioural metrics of interest. The metrics encompass the main metrics of interest and also the supplementary additional metrics not considered in the main full analysis (non‐dominant hand grip strength, 2‐min walk endurance test, 4‐m walk gait speed test). Lower triangle contains Pearson's correlation coefficients, with yellow‐green scale to improve clarity. Absolute correlation coefficient values below 0.3 are provided in grey‐coloured numbers, above 0.3 in black colour. The upper triangle contains False Discovery Rate corrected *p*‐values. See also Table 1 and Table S3 for the number of available metrics.
**Table S2:** Basic demographic and clinical characteristics of the cohort classified based on positive or negative clinical evaluation of 18F‐florbetaben PET scans. Data presented as average (standard deviation) and number of subjects with available datapoints, unless specified otherwise. *p* value for two‐tailed two‐sample Student's *t* test (for continuous variables) or χ2 (for categorical variables) after False Discovery Rate (FDR) correction is presented.
**Table S3:** β‐amyloid load over predetermined regions of interest, expressed as florbetaben standardised uptake value ratios (average [standard deviation]), their association with age based on Pearson's correlation coefficient, including False Discovery Rate (FDR) corrected *p*‐values. Furthermore, the green part of the table contains group averages (standard deviations) of voxel‐wise Pearson's correlation coefficients of β‐amyloid load with amyloid precursor protein (APP) messenger RNA expression intensity, as depicted in the Figure 2 in the main text.
**Table S4:** Correlation of β‐amyloid standardised uptake value ratios (SUVRs) with demographic and clinical metrics over preselected regions of interest. Results provided as Pearson's correlation coefficients (False Discovery Rate corrected *p*‐values), green bold accentuation marks statistically significant results. BMI, body mass index.
**Table S5:** Supporting Information for the extended clinical correlation analysis on further physical fitness parameters previously excluded from the analysis due to low number of subjects. Group averages (standard deviations), including the number of subjects with available data is provided, followed by the correlation of βamyloid standardised uptake value ratios (SUVRs) with relevant clinical metrics over preselected regions of interest. Results provided as Pearson's correlation coefficients, green bold accentuation marks statistically significant results. No formal testing of statistical significance was performed due to the exploratory nature of the analysis and limited sample sizes for gait tests.
**Table S6:** Voxel‐wise correlation of β‐amyloid standardised uptake value ratios (SUVRs) with magnetic resonance imaging (MRI) metrics over preselected regions of interest. Results provided as cross‐sectional cohort average voxel‐wise Pearson's correlation coefficients [cross‐sectional standard deviations of individual voxelwise Pearson's correlation coefficients]. False Discovery Rate corrected *p*‐values, green bold accentuation marks statistically significant results (see Table S7). CBF, cerebral blood flow; DeCe, weighted degree centrality; FA, fractional anisotropy; fALFF, fractional amplitude of low‐frequency fluctuations; IVF, intracellular volume fraction; MD, mean diffusivity; ODI, orientation dispersion index; ReHo, regional homogeneity; T1w/T2w, T1‐weighted/T2‐weighed ratio; WVF, free water volume fraction.
**Table S7:** Statistical significance of voxel‐wise correlation of β‐amyloid standardised uptake value ratios (SUVRs) with magnetic resonance imaging (MRI) metrics over preselected regions of interest. Results presented as permutation‐based one‐way one‐sample *T* value (False Discovery Rate corrected *p*‐value) evaluating whether absolute values of subject‐specific, Fisher transformed voxel‐wise Pearson's correlation coefficients are statistically significantly higher than 0.3 preselected as the empirical medium effect size (Cohen 1988). Green bold accentuation marks statistically significant results. CBF, cerebral blood flow; DeCe, weighted degree centrality; FA, fractional anisotropy; fALFF, fractional amplitude of low‐frequency fluctuations; IVF, intracellular volume fraction; MD, mean diffusivity; ODI, orientation dispersion index; ReHo, regional homogeneity; T1w/T2w, T1‐weighted/T2‐weighed ratio; WVF, free water volume fraction.
**Table S8:** Correlation matrices of demographic and clinical metrics over preselected regions of interest for individual Magnetic resonance imaging (MRI) metrics, to be utilised for the comparison of clinical similarity assessment between β‐amyloid SUVR maps and MRI metrics. Results provided as Pearson's correlation coefficients, with yellow‐green scale to improve clarity. No formal testing of statistical significance was performed due to the intermediate nature of the analysis. Statistical significance testing, including multiple comparison correction provided at the level of whole Aim 3 (see also the row “Clinical similarity” in Figure 3B in the main text). BMI, body mass index; post. cing., posterior cingulate.

## Data Availability

HCP‐A datasets and standard processing scripts are publicly available; for further details see https://www.humanconnectome.org/.
