# Supplementary material for: Clinical and MRI Correlates of β‐Amyloid Load Inconsistent With Its Presumed Neurotoxicity in Cognitively Healthy Ageing
Source: J Neurochem. 2025 Sep 23;169(9):e70241. doi: 10.1111/jnc.70241 (PMC12455269; doi:10.1111/jnc.70241)
Supplement: Supplementary file 1 — Data S1: Flowchart documenting the pre‐screening process from the full HCP‐A database acquired at the University of Minnesota, USA from 2017 to 2022, stating relevant numbers of individuals and reasons for exclusion. “Declined participation” includes HCP‐A participants who explicitly declined the option to be contacted for follow‐up projects and HCP‐A participants who declined after being contacted with the offer for participation in this specific study. MoCA, Montreal Cognitive Assessment; MRS, magnetic resonance spectroscopy; TICS, Telephone Interview for Cognitive Status (the full study protocol included also MRS data which is not the subject of this analysis). Figure S2: Amyloid precursor protein (APP) transcriptome atlas with cortical reconstruction at the left side with overlaid Human Connectome Project (HCP) cortical parcellation borders (Glasser et al. 2016) and subcortical grey matter at the right side over a T1‐weighted template. Colour scales represent log2 messenger ribonucleic acid expression intensity. Subcortical structures shown in 6 slices z = 23, 11, −1, −13, −25, −37 (Montreal Neurological Institute coordinate system) with focus on subcortical grey matter structures, since no white matter expression intensity data was available. High APP expression intensities depicted in lateral temporal cortices, anterior and posterior cingulate, precuneus, but also lateral parts of both putamina. On the other hand, low APP expression intensities are present in the right temporal cortex, lateral frontal cortices and the cerebellum. Laterality convention where the right side of the figure corresponds to the right side of the brain is used. See Table S4 for further anatomical and statistical information on significant regions. L, left; R, right. Figure S3: Regions of interest in a “representative” subject. (A) Native resolution of the T1‐weighted space; (B) 2‐mm isotropic voxel resolution thresholded with 0.9 inclusion probability to mitigate partial volume effect [file JNC-169-0-s001.pdf]

Clinical and MRI correlates of β-amyloid load inconsistent with its presumed neurotoxicity in cognitively healthy ageing

Pavel Filip[1,2,3], J. Riley McCarten[4,5], Laura Hemmy[4,6], Jillian Crocker[1], Michael Wolf[1], Jeromy Thotland[1], Zuzan Cayci[7], Todd Kes[7], Shalom Michaeli[1], Melissa Terpstra[1], Silvia Mangia[1*]

[1] Center for Magnetic Resonance Research (CMRR), University of Minnesota, Minneapolis, MN, USA

[2] Department of Neurology, Charles University, First Faculty of Medicine and General University Hospital, Prague, Czech Republic

[3] Department of Cybernetics, Czech Technical University in Prague, Prague, Czech Republic

[4]Geriatric Research, Education and Clinical Center, Veterans Affairs Medical Center, Minneapolis, MN, USA

[5]Department of Neurology, University of Minnesota Medical School, Minneapolis, MN, USA

[6]Department of Psychiatry, University of Minnesota Medical School, Minneapolis, MN, USA

[7]Department of Radiology, University of Minnesota, Minneapolis, MN, USA

* Corresponding author: Silvia Mangia
postal address: Center for Magnetic Resonance Research, University of Minnesota, 2021 Sixth St. SE, Minneapolis, MN 55455, USA.
email: mangia@umn.edu

Supplementary methods
PET acquisition and evaluation

The study utilised 18F-florbetaben as ß-amyloid radiotracer. Its delivery to the PET department of the Center for Clinical Imaging and Research (CCIR) strictly followed all applicable regulatory guidelines. Eligible participants received a single dose of 8.1 mCi ± 10% 18F-florbetaben in a maximum volume of 10 ml, administered as a slow intravenous bolus, followed by a 10 ml saline flush. Sixty minutes thereafter, a brain PET/CT scan was performed using a Siemens Biograph 64 scanner, with the following parameters: static PET acquisition duration 20 minutes, 120 KeV, 350 mA (reference value, dose modulation for each participant as calculated by Siemens CareDose4D software was utilised), slice thickness of 3.00 mm, field of view 250 mm^2, and a matrix size of 256 × 256. Participants' heads were immobilised using a head holder and fixation devices to minimise motion artefacts.

All PET datasets were first corrected for radioactive decay, detector dead time, measured attenuation, and scatter. They were subsequently reconstructed with a time-of-flight–enabled iterative algorithm (4 iterations, 21 subsets) into a 128 × 128 × 128 matrix with a zoom factor of 2.0, followed by Gaussian post-smoothing with a 3.0 mm full-width at half-maximum filter. Florbetaben PET images were visually interpreted by an experienced brain PET reader (ZC, 16 years of experience) blinded to the clinical diagnosis and all other clinical details. The standard clinical assessment procedure was based on purely visual inspection of axial scans in greyscale palette using a predefined Regional Cortical Tracer Uptake (RCTU) scoring system (1 = no tracer uptake, 2 = moderate tracer uptake, and 3 = pronounced tracer uptake). The cortical regions considered include the lateral temporal cortex, frontal cortex, parietal cortex, and the posterior cingulate cortex/precuneus. Brain Amyloid Plaque Load (BAPL) score was considered the final outcome of clinical evaluation. If all 4 regions show RCTU score of 1, BAPL score is classified as "1" (β-amyloid negative scan); if one or more regions shows RCTU score of 2 and no region of RCTU 3, BAPL score is classified as "2" (β-amyloid positive scan with moderate amyloid plaque deposition). If one or more regions shows RCTU score of 3, BAPL score is classified as "3" (β-amyloid positive scan with pronounced amyloid deposition).

References:

Piramal Imaging Limited. Summary of Product Characteristics, 4th ed.; Piramal Imaging Limited: Boston, MA, USA, 2014.

Sabri, O.; Seibyl, J.; Rowe, C.; Barthel, H. Beta-amyloid imaging with florbetaben. Clin. Transl. Imaging 2015, 3, 13–26

Supplementary figures

Supplementary figure 1: Flowchart documenting the pre-screening process from the full HCP-A database acquired at the University of Minnesota, USA from 2017 to 2022, stating relevant numbers of individuals and reasons for exclusion. "Declined participation" includes HCP-A participants who explicitly declined the option to be contacted for follow-up projects and HCP-A participants who declined after being contacted with the offer for participation in this specific study. Abbreviations: MoCA – Montreal Cognitive Assessment; TICS – Telephone Interview for Cognitive Status; MRS – Magnetic Resonance Spectroscopy (the full study protocol included also MRS data which is not the subject of this analysis).

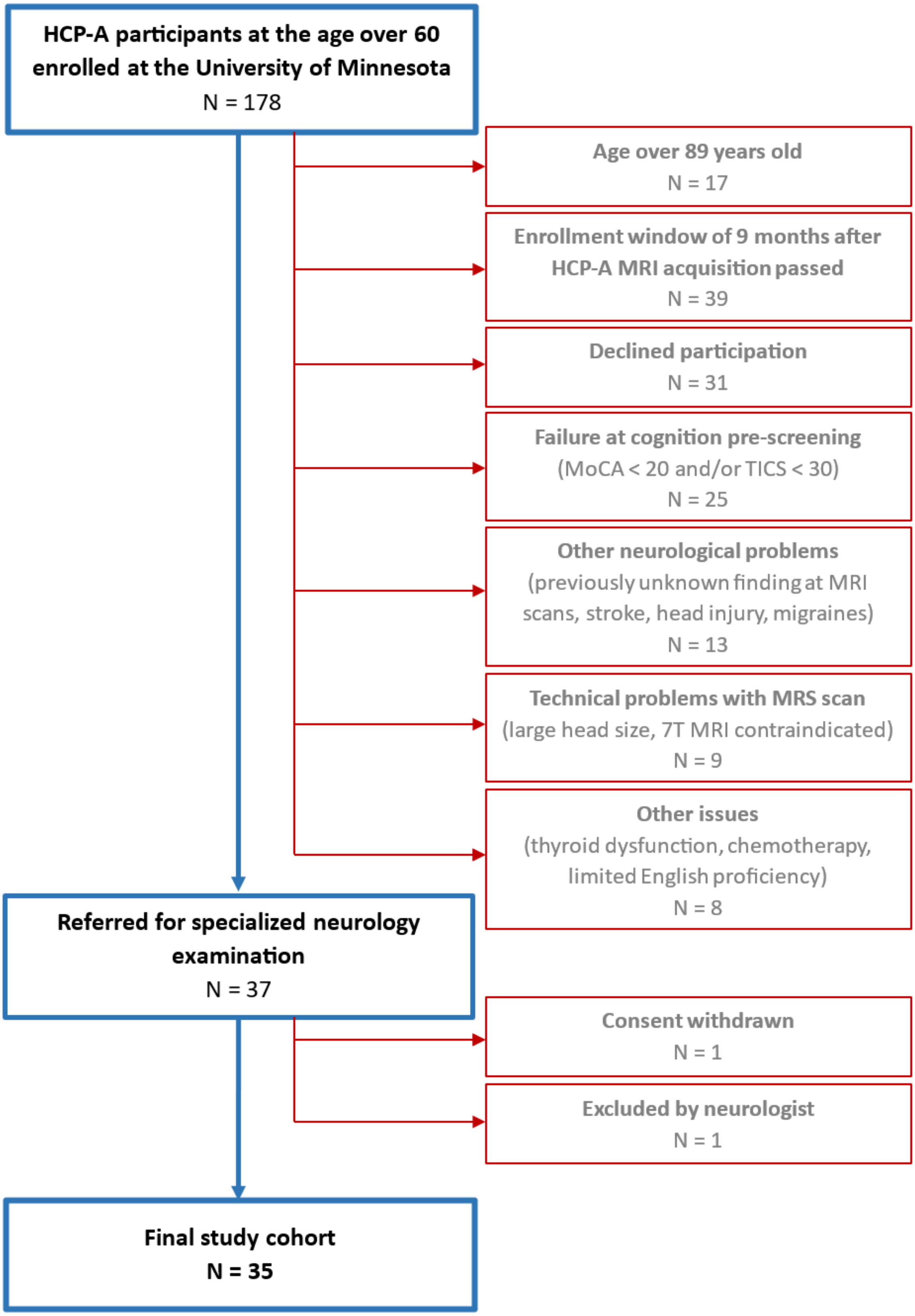

Supplementary figure 2: Amyloid precursor protein (APP) transcriptome atlas with cortical reconstruction at the left side with overlaid Human Connectome Project (HCP) cortical parcellation borders [Glasser et al., 2016] and subcortical grey matter at the right side over a T1-weighted template. Colour scales represent $\log_2$ messenger ribonucleic acid expression intensity. Subcortical structures shown in 6 slices z = 23, 11, -1, -13, -25, -37 (Montreal Neurological Institute coordinate system) with focus on subcortical grey matter structures, since no white matter expression intensity data was available. High APP expression intensities depicted in lateral temporal cortices, anterior and posterior cingulate, precuneus, but also lateral parts of both putamina. On the other hand, low APP expression intensities are present in the right temporal cortex, lateral frontal cortices and the cerebellum. Laterality convention where the right side of the figure corresponds to the right side of the brain is used. See Supplementary table 4 for further anatomical and statistical information on significant regions. Abbreviations: L – left; R – right.

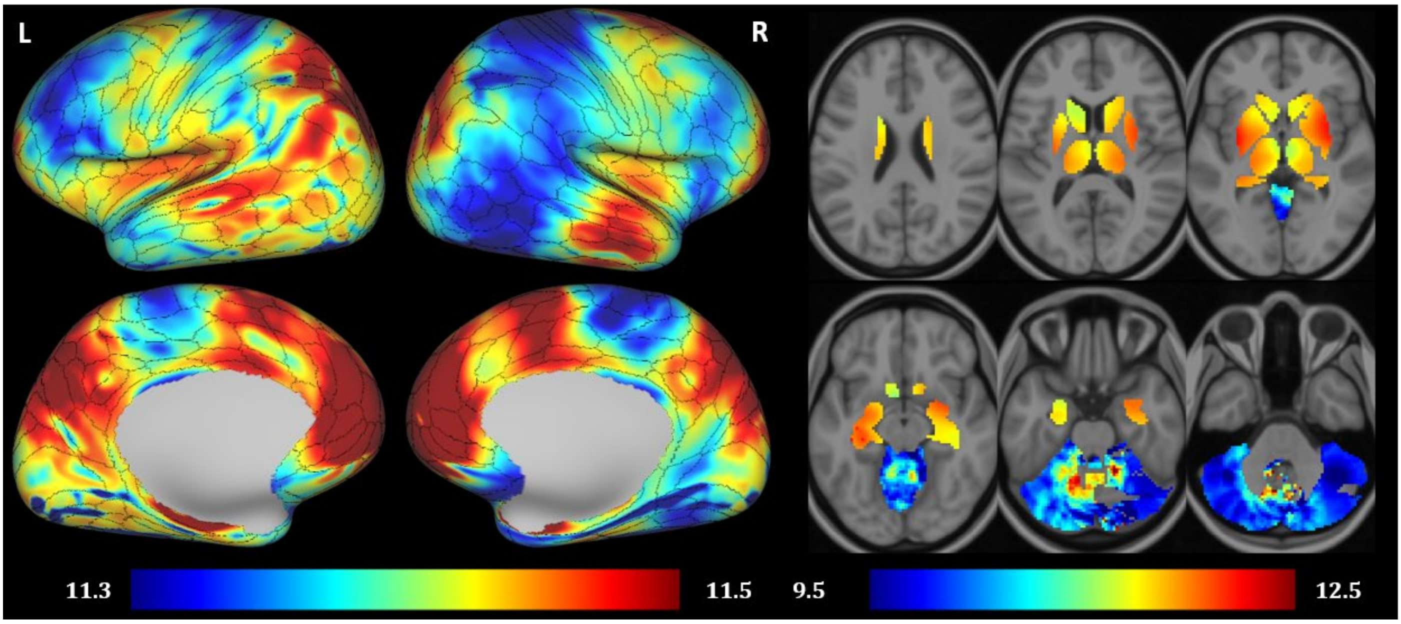

Supplementary figure 3: Regions of interest in a "representative" subject. **A) Native resolution** of the T1-weighted space; **B) 2-mm isotropic voxel resolution** thresholded with 0.9 inclusion probability to mitigate partial volume effects in lower resolution scans as resting-state functional MRI, cerebral blood flow and diffusion-weighted imaging metrics. Colour coding as follows: entorhinal cortex – red; subcortical limbic structures – orange; temporal lobe cortex – yellow; anterior cingulate – cyan; posterior cingulate and precuneus – blue; basal ganglia – green. Whole cortex and whole white matter masks not depicted for clarity purposes. Z coordinates of slices 40, 34, 27, 21, 14, 18, 2, -5, -11, -18, -24, -30

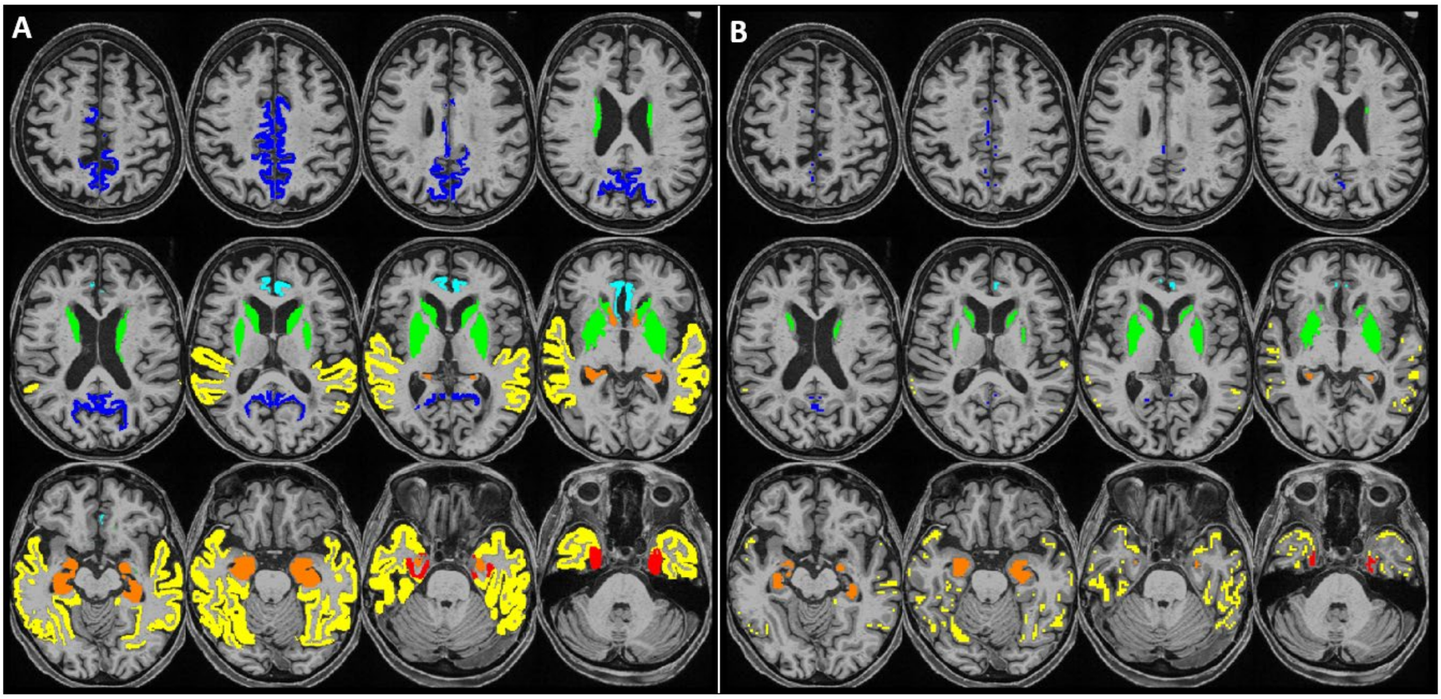

Supplementary figure 4: Average β-amyloid standardised uptake value ratio (SUVR) map separately over the full cohort (35 participants), individuals with negative (24 participants) and positive clinical reading (11 individuals): cortical surface reconstruction at the left side with overlaid Human Connectome Project (HCP) cortical parcellation borders [Glasser et al., 2016] and subcortical structures in Montreal Neurological Institute (MNI) space at the right side shown in 6 slices z = 48, 29, 10, -28, -47 (MNI coordinate system). Colour-coding provided in scales below. Laterality convention where the right side of the figure corresponds to the right side of the brain is used.

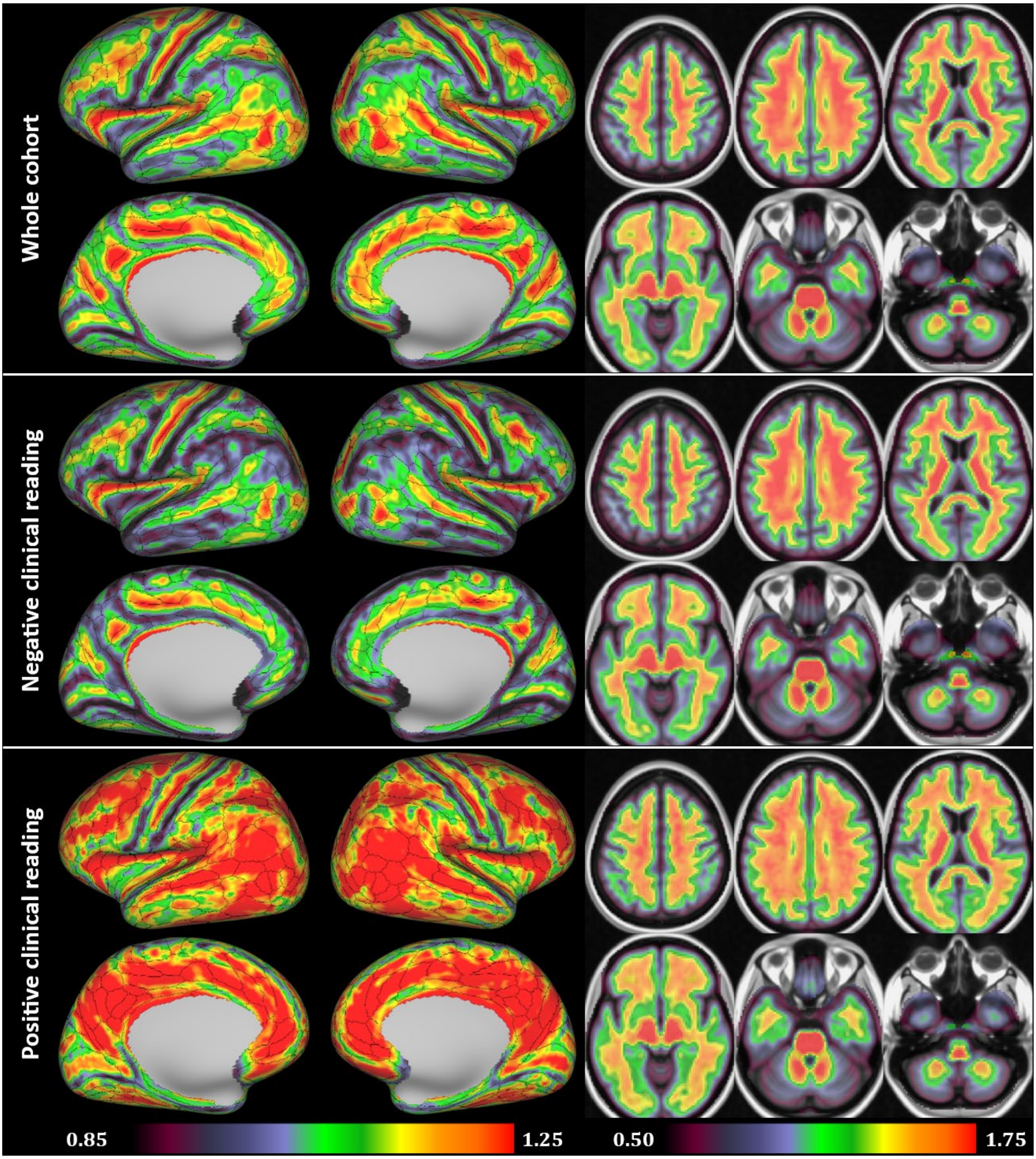

Supplementary tables

Supplementary table 1: Cross-correlation matrix of individual demographic and behavioural metrics of interest. The metrics encompass the main metrics of interest and also the supplementary additional metrics not considered in the main full analysis (non-dominant hand grip strength, 2-minute walk endurance test, 4-meter walk gait speed test). Lower triangle contains Pearson's correlation coefficients, with yellow-green scale to improve clarity. Absolute correlation coefficient values below 0.3 are provided in grey-coloured numbers, above 0.3 in black colour. The upper triangle contains False Discovery Rate corrected p-values. See also Table 1 and Supplementary table 3 for the number of available metrics.

	Age	BMI	Fluid cognition	Crystallized cognition	Grip strength, dominant hand	Grip strength, non-dominant hand	2-minute walk endurance test	4-Meter Walk Gait Speed Test
**Age** [years]		0.119	0.219	0.121	0.552	0.545	0.240	0.147
**BMI** [kg.m^{-2}]	0.302		0.157	0.463	0.467	0.478	0.170	0.290
**Fluid cognition** [age-corrected standard score]	-0.208	-0.258		0.077	0.066	0.132	0.131	0.442
**Crystallized cognition** [age-corrected standard score]	-0.331	-0.076	0.413		0.178	0.369	0.083	0.095
**Grip strength, dominant hand** [age-corrected standard score]	0.004	-0.055	0.424	0.249		0.000	0.118	0.420
**Grip strength, non-dominant hand** [age-corrected standard score]	0.015	0.066	0.321	0.137	0.826		0.535	0.482
**2-minute walk endurance test** [age-corrected standard score]	-0.267	-0.338	0.452	0.569	0.405	0.004		0.145
**4-meter walk gait speed test** [m.s^{-1}]	-0.404	-0.223	0.121	0.482	0.141	0.072	0.374	

Supplementary table 2: Basic demographic and clinical characteristics of the cohort classified based on positive or negative clinical evaluation of 18F-florbetaben PET scans. Data presented as average [standard deviation] and number of subjects with available datapoints, unless specified otherwise. P value for two-tailed two-sample Student's T test (for continuous variables) or $\chi 2$ (for categorical variables) after False Discovery Rate (FDR) correction is presented.

	Negative evaluation	Positive evaluation	Number of subjects with available data	FDR-corrected p value
**Age** [years]	71.79 [8.58]	76.18 [9.78]	24/11	0.329
**Sex** [females/males]	18 / 6	4 / 7	24/11	0.128
**Body mass index (BMI)** [kg.m^{-2}]	25.42 [3.87]	29.05 [4.81]	24/11	0.096
**Education** [years]	16.17 [2.04]	16.64 [2.69]	24/11	0.465
**BAPL score** [number of subjects with score 1/2/3]	24 / 0 / 0	0 / 6 / 5		
**NIH Toolbox Cognitive Function Battery (CFB)**				
Fluid cognitive abilities [age-corrected standard score]	112.46 [15.36]	113.27 [15.81]	24/11	0.576
Crystallized cognitive abilities [age-corrected standard score]	109.78 [11.14]	110.00 [12.89]	23/11	0.547
**NIH Toolbox Motor Domain**				
Grip strength, dominant hand [age-corrected standard score]	100.05 [15.46]	106.11 [15.58]	22/9	0.309
2-minute walk endurance test [age-corrected standard score]	114.00 [10.76]	120.33 [10.89]	13/6	0.301

Supplementary table 3: ß-amyloid load over predetermined regions of interest, expressed as florbetaben standardised uptake value ratios (average [standard deviation]), their association with age based on Pearson's correlation coefficient, including False Discovery Rate (FDR) corrected p-values. Furthermore, the green part of the table contains group averages [standard deviations] of voxel-wise Pearson's correlation coefficients of ß-amyloid load with amyloid precursor protein (APP) messenger RNA expression intensity, as depicted in the Figure 2 in the main text.

Area of interest	Florbetaben SUVRs	Association with age		Florbetaben SUVRs vs APP mRNA expression intensity
		Pearson's r	FDR corrected p-value	
**Entorhinal cortex**	0.895 [0.077]	0.231	0.132	**0.355 [0.078]**
**Limbic system**	0.987 [0.057]	-0.003	0.327	-0.077 [0.052]
**Temporal lobe**	0.954 [0.135]	0.288	0.108	0.147 [0.051]
**Anterior cingulate**	0.972 [0.212]	0.201	0.136	-0.252 [0.078]
**Posterior cingulate + precuneus**	1.008 [0.205]	0.249	0.116	-0.153 [0.035]
**Whole cortex**	0.975 [0.144]	0.268	0.107	0.001 [0.044]
**Basal ganglia**	1.154 [0.097]	0.058	0.268	**-0.381 [0.071]**

Supplementary table 4: Correlation of ß-amyloid standardised uptake value ratios (SUVRs) with demographic and clinical metrics over preselected regions of interest. Results provided as Pearson's correlation coefficients [False Discovery Rate corrected p-values], green bold accentuation marks statistically significant results.
Abbreviations: BMI – body mass index.

	Age	BMI	Grip strength	Crystallized cognition	Fluid cognition
**Entorhinal cortex**	0.231 [0.132]	**0.400 [0.040]**	0.344 [0.096]	0.095 [0.237]	0.063 [0.277]
**Limbic system**	-0.003 [0.327]	0.316 [0.094]	0.108 [0.241]	-0.022 [0.308]	-0.284 [0.104]
**Temporal lobe**	0.288 [0.108]	**0.412 [0.041]**	0.216 [0.148]	0.168 [0.159]	0.062 [0.271]
**Anterior cingulate**	0.201 [0.136]	**0.444 [0.029]**	0.203 [0.145]	0.206 [0.141]	0.108 [0.239]
**Posterior cingulate + precuneus**	0.249 [0.116]	0.468 [0.053]	0.271 [0.116]	0.177 [0.154]	0.295 [0.110]
**Whole cortex**	0.268 [0.107]	**0.465 [0.028]**	0.220 [0.151]	0.192 [0.140]	0.095 [0.243]
**Basal ganglia**	0.058 [0.268]	0.357 [0.068]	0.295 [0.104]	0.226 [0.135]	0.022 [0.317]

Supplementary table 5: Supplementary data for the extended clinical correlation analysis on further physical fitness parameters previously excluded from the analysis due to low number of subjects. Group averages [standard deviations], including the nubmer of subjects with available data is providded, followed by the correlation of ß-amyloid standardised uptake value ratios (SUVRs) with relevant clinical metrics over preselected regions of interest. Results provided as Pearson's correlation coefficients, green bold accentuation marks statistically significant results. No formal testing of statistical significance was performed due to the exploratory nature of the analysis and limited sample sizes for gait tests.

Metric of interest	Average [standard deviation]	Number of subjects with available data	Pearson's correlation coefficient with SUVRs over regions of interest						
			Entorhinal cortex	Limbic system	Temporal lobe	Anterior cingulate	Posterior cingulate + precuneus	Whole cortex	Basal ganglia
**Grip strength, non-dominant hand** [age-corrected standard score]	100.194 [15.404]	31	0.241	0.088	0.049	0.033	0.061	0.054	0.099
**2-minute walk endurance test** [age-corrected standard score]	116.000 [10.924]	19	-0.089	-0.055	-0.002	0.014	0.064	0.019	0.155
**4-meter walk gait speed test** [m.s^{-1}]	1.166 [0.150]	20	0.201	0.354	0.155	0.197	0.102	0.185	0.391

Supplementary table 6: Voxel-wise correlation of ß-amyloid standardised uptake value ratios (SUVRs) with magnetic resonance imaging (MRI) metrics over preselected regions of interest. Results provided as cross-sectional cohort average voxel-wise Pearson's correlation coefficients [cross-sectional standard deviations of individual voxel-wise Pearson's correlation coefficients]. False Discovery Rate corrected p-values], green bold accentuation marks statistically significant results (see Supplementary table 7).

Abbreviations: T1w/T2w – T1-weighted/T2-weighed ratio; IVF – intracellular volume fraction; WVF – free water volume fraction; ODI – orientation dispersion index; FA – fractional anisotropy; MD – mean diffusivity, CBF – cerebral blood flow; DeCe – weighted degree centrality; fALFF – fractional amplitude of low-frequency fluctuations; ReHo – regional homogeneity.

	T1w/T2w	IVF	WVF	ODI	FA	MD	CBF	wDeCe	fALFF	ReHo
**Entorhinal cortex**	0.036 [0.330]	-0.278 [0.369]	-0.342 [0.315]	0.119 [0.450]	**0.385 [0.237]**	-0.142 [0.377]	0.382 [0.241]	0.160 [0.093]	0.192 [0.136]	0.138 [0.141]
**Limbic system**	**0.492 [0.147]**	-0.046 [0.153]	-0.142 [0.134]	**-0.633 [0.128]**	**0.676 [0.105]**	-0.176 [0.142]	0.050 [0.117]	-0.027 [0.024]	-0.180 [0.069]	-0.204 [0.068]
**Temporal lobe**	-0.023 [0.211]	-0.100 [0.175]	**-0.434 [0.120]**	0.125 [0.230]	**0.583 [0.143]**	-0.095 [0.195]	**0.439 [0.098]**	0.135 [0.021]	0.147 [0.057]	0.135 [0.059]
**Anterior cingulate**	**0.644 [0.142]**	**-0.617 [0.227]**	**-0.750 [0.160]**	**-0.473 [0.239]**	**0.557 [0.225]**	**-0.750 [0.175]**	0.253 [0.240]	0.112 [0.062]	0.185 [0.144]	0.159 [0.141]
**Post. cing. + precuneus**	**0.669 [0.188]**	-0.309 [0.292]	**-0.642 [0.111]**	**-0.536 [0.218]**	**0.584 [0.138]**	**-0.669 [0.132]**	0.156 [0.197]	0.117 [0.035]	0.112 [0.062]	0.089 [0.069]
**Whole cortex**	0.179 [0.176]	-0.168 [0.120]	**-0.500 [0.088]**	-0.040 [0.201]	**0.585 [0.148]**	-0.277 [0.145]	**0.357 [0.100]**	0.063 [0.022]	0.050 [0.056]	0.067 [0.054]
**Basal ganglia**	**0.693 [0.182]**	0.102 [0.184]	-0.360 [0.371]	**-0.668 [0.099]**	**0.825 [0.078]**	**-0.496 [0.292]**	0.054 [0.225]	-0.227 [0.023]	**-0.472 [0.071]**	**-0.356 [0.085]**
**White matter**	**0.685 [0.065]**	0.093 [0.183]	**-0.607 [0.115]**	**-0.390 [0.121]**	**0.439 [0.092]**	**-0.736 [0.079]**	-0.163 [0.098]			

Supplementary table 7: Statistical significance of voxel-wise correlation of ß-amyloid standardised uptake value ratios (SUVRs) with magnetic resonance imaging (MRI) metrics over preselected regions of interest. Results presented as permutation-based one-way one-sample T value [False Discovery Rate corrected p-value] evaluating whether absolute values of subject-specific, Fisher transformed voxel-wise Pearson's correlation coefficients are statistically significantly higher than 0.3 preselected as the empirical medium effect size (Cohen, 1988). Green bold accentuation marks statistically significant results.

Abbreviations: T1w/T2w – T1-weighted/T2-weighed ratio; IVF – intracellular volume fraction; WVF – free water volume fraction; ODI – orientation dispersion index; FA – fractional anisotropy; MD – mean diffusivity, CBF – cerebral blood flow; DeCe – weighted degree centrality; fALFF – fractional amplitude of low-frequency fluctuations; ReHo – regional homogeneity.

	T1w/T2w	IVF	WVF	ODI	FA	MD	CBF	wDeCe	fALFF	ReHo
Entorhinal cortex	-4.375 [>0.500]	-0.162 [>0.500]	0.986 [0.347]	-2.193 [>0.500]	**2.301 [0.036]**	-2.265 [>0.500]	1.889 [0.081]	-7.959 [>0.500]	-4.192 [>0.500]	-6.240 [>0.500]
Limbic system	**8.544 [<0.001]**	-9.229 [>0.500]	-6.275 [>0.500]	**11.965 [<0.001]**	**14.474 [<0.001]**	-4.523 [>0.500]	-12.078 [>0.500]	-36.873 [>0.500]	-6.747 [>0.500]	-6.143 [>0.500]
Temporal lobe	-7.205 [>0.500]	-6.236 [>0.500]	**5.996 [<0.001]**	-4.137 [>0.500]	**9.832 [<0.001]**	-5.764 [>0.500]	**5.652 [<0.001]**	-26.559 [>0.500]	-12.225 [>0.500]	-13.188 [>0.500]
Anterior cingulate	**15.191 [<0.001]**	**7.667 [<0.001]**	**13.150 [<0.001]**	**4.260 [0.001]**	**6.415 [<0.001]**	**12.425 [<0.001]**	-1.025 [>0.500]	-16.172 [>0.500]	-4.178 [>0.500]	-5.437 [>0.500]
Post. cing. + precuneus	**12.275 [<0.001]**	0.412 [>0.500]	**13.221 [<0.001]**	**6.125 [<0.001]**	**10.089 [<0.001]**	**12.684 [<0.001]**	-4.040 [>0.500]	-26.888 [>0.500]	-13.389 [>0.500]	-13.850 [>0.500]
Whole cortex	-3.397 [>0.500]	-5.673 [>0.500]	**10.062 [<0.001]**	-7.193 [>0.500]	**9.708 [<0.001]**	-0.436 [>0.500]	**2.623 [0.014]**	-48.254 [>0.500]	-21.758 [>0.500]	-21.795 [>0.500]
Basal ganglia	**13.401 [<0.001]**	-5.872 [>0.500]	1.114 [0.294]	**14.644 [<0.001]**	**19.358 [<0.001]**	**3.978 [0.001]**	-6.185 [>0.500]	-6.194 [>0.500]	**8.480 [<0.001]**	**3.139 [0.003]**
White matter	**36.863 [<0.001]**	-6.174 [>0.500]	**11.985 [<0.001]**	**4.316 [<0.001]**	**7.382 [<0.001]**	**17.494 [<0.001]**	-7.333 [>0.500]			
Clinical similarity	**0.589 [0.002]**	0.341 [0.080]	0.123 [0.531]	-0.280 [0.150]	**0.533 [0.007]**	-0.031 [0.854]	-0.374 [0.066]	-0.288 [0.150]	**-0.415 [0.044]**	-0.256 [0.165]

Supplementary table 8: Correlation matrices of demographic and clinical metrics over preselected regions of interest for individual Magnetic resonance imaging (MRI) metrics, to be utilised for the comparison of clinical similarity assessment between ß-amyloid SUVR maps and MRI metrics. Results provided as Pearson's correlation coefficients, with yellow-green scale to improve clarity. No formal testing of statistical significance was performed due to the intermediate nature of the analysis. Statistical significance testing, including multiple comparison correction provided at the level of whole Aim 3 (see also the row "Clinical similarity" in Figure 3B in the main text). Abbreviations: BMI - body mass index; post. cing. - posterior cingulate

Metric	Parameter	Entorhinal cortex	Limbic system	Temporal lobe	Anterior cingulate	Post. cing. + precuneus	Whole cortex	Basal ganglia
**T1-weighted/T2-weighed ratio**	Age	-0.2016	-0.3138	0.0512	0.2271	-0.0167	0.1838	-0.1200
	BMI	-0.0703	-0.0263	0.1946	0.1381	0.2828	0.2249	-0.0522
	Grip strength	-0.0794	-0.2207	-0.3176	0.0643	-0.2780	-0.1910	-0.1863
	Crystallized cognition	0.0143	0.0473	-0.2435	-0.0301	-0.0756	-0.1518	-0.0037
	Fluid cognition	-0.3227	-0.2832	-0.4133	-0.2209	-0.2398	-0.3186	-0.2832
**Fraction of intracellular water**	Age	0.0790	0.0109	-0.2255	0.0825	0.0397	-0.0524	0.4489
	BMI	0.3818	0.2283	0.2757	0.1626	0.2279	0.2431	0.3148
	Grip strength	-0.0736	0.2667	0.0742	-0.1466	0.1131	0.2147	-0.2233
	Crystallized cognition	0.1828	0.0086	0.2554	0.0147	0.1891	0.1857	-0.1996
	Fluid cognition	-0.1338	-0.1039	-0.0300	-0.2402	0.0623	-0.0027	-0.2059
**Fraction of isotropic water (Free water fraction)**	Age	0.4322	0.4985	0.2704	0.2944	0.4444	0.3645	0.7300
	BMI	0.0951	0.1531	0.4102	0.1661	0.1573	0.2883	0.3233
	Grip strength	-0.2671	0.1843	-0.0253	-0.1104	-0.0990	0.0110	-0.0580
	Crystallized cognition	-0.2869	-0.1465	0.0198	-0.0866	-0.1604	-0.0564	-0.1432
	Fluid cognition	-0.1931	0.1413	-0.0987	-0.1171	-0.1897	-0.1347	0.0788
**Orientation dispersion index**	Age	-0.1121	-0.0307	-0.5723	-0.0867	-0.4653	-0.6340	-0.1828
	BMI	0.0663	0.1384	-0.0406	-0.0943	-0.1715	-0.1546	0.0037
	Grip strength	-0.1553	0.2233	-0.2291	-0.0681	-0.2231	-0.2766	-0.3863
	Crystallized cognition	0.0530	0.1793	0.2138	-0.0718	0.1266	0.1445	-0.3079
	Fluid cognition	-0.0701	0.1103	-0.0701	-0.0141	0.0113	-0.0679	-0.1840
**Fractional anisotropy**	Age	-0.1092	0.0226	0.4967	0.2256	0.4695	0.6114	0.5201
	BMI	0.1649	0.2132	0.3157	0.2369	0.3224	0.3508	0.3337
	Grip strength	0.1662	0.0293	0.3287	-0.0064	0.2233	0.3447	0.1365
	Crystallized cognition	0.0764	-0.1053	-0.0753	-0.0297	-0.1071	-0.1624	-0.0796
	Fluid cognition	-0.1083	-0.1799	0.0357	-0.2232	-0.0060	0.0091	-0.1754
**Mean diffusivity**	Age	0.3207	0.5419	0.4106	0.0904	0.3262	0.3513	0.4785
	BMI	0.0551	0.0847	0.2115	0.0918	0.1060	0.1602	0.1468
	Grip strength	-0.1537	0.0744	0.0323	0.1296	-0.1219	-0.1151	0.0531
	Crystallized cognition	-0.2365	-0.1444	-0.0536	-0.0506	-0.1818	-0.1155	0.0506
	Fluid cognition	-0.1334	0.2006	0.0302	0.1624	-0.1745	-0.0974	0.2210
**Cerebral blood perfusion**	Age	-0.1450	-0.4036	-0.5206	-0.3595	-0.5247	-0.5286	-0.4584
	BMI	-0.0106	-0.3314	-0.3726	-0.1072	-0.3563	-0.3340	-0.1839
	Grip strength	-0.2460	-0.1132	-0.2095	-0.0633	-0.2396	-0.2407	-0.0703
	Crystallized cognition	-0.3030	0.0353	0.0570	0.0852	0.0122	0.0562	0.0089
	Fluid cognition	-0.1242	0.1882	0.0416	-0.0137	0.0284	0.0129	-0.0183
**Weighed degree centrality**	Age	0.3216	0.0338	-0.0176	-0.1398	-0.0726	-0.2562	0.3284
	BMI	0.2415	-0.0167	-0.3895	-0.0720	-0.0905	-0.4108	-0.0712
	Grip strength	0.0334	-0.0860	-0.2323	-0.1189	-0.3085	-0.3138	-0.0248
	Crystallized cognition	-0.3245	0.2062	-0.0760	0.1808	-0.0190	0.2535	-0.1287
	Fluid cognition	-0.0734	0.0120	-0.0326	0.0797	-0.0970	-0.0776	-0.0215
**Fractional amplitude of low frequency fluctuations**	Age	-0.1022	-0.2351	-0.1893	-0.2264	-0.3283	-0.3124	-0.4083
	BMI	-0.1421	-0.0320	-0.2586	-0.0901	-0.1820	-0.2207	-0.1613
	Grip strength	0.0640	0.1437	0.0323	-0.1325	0.0174	0.0563	0.1589
	Crystallized cognition	0.3830	0.4104	0.2804	0.0259	0.1316	0.3169	0.3689
	Fluid cognition	0.0370	0.1665	0.0280	0.0915	-0.0320	0.1075	0.2299
**Regional homogeneity**	Age	0.1124	-0.0803	0.0289	-0.0448	0.0172	-0.1333	-0.1982
	BMI	0.0530	0.0768	-0.0840	0.0283	-0.1678	-0.1381	-0.1171
	Grip strength	0.0131	0.2293	-0.0310	-0.0553	-0.1685	-0.0642	-0.0017
	Crystallized cognition	0.3632	0.3152	0.2182	0.1169	-0.0656	0.2726	0.4150
	Fluid cognition	-0.1426	0.0421	-0.0924	0.0208	-0.0926	0.0344	0.0757
